# Evaluation of the psychoemotional status of young adults with symptoms of temporomandibular disorders

**DOI:** 10.1002/brb3.1443

**Published:** 2019-10-15

**Authors:** Monika Maślak‐Bereś, Jolanta E. Loster, Aneta Wieczorek, Bartłomiej W. Loster

**Affiliations:** ^1^ Prosthodontic Department Dental Institute Medical College Jagiellonian University in Kraków Kraków Poland; ^2^ Orthodontic Department Dental Institute Medical College Jagiellonian University in Kraków Kraków Poland

**Keywords:** Beck's depression inventory, PSS‐10 Perceived Stress Scale, psychoemotional status, research diagnostic criteria for temporomandibular disorders, temporomandibular disorders

## Abstract

**Background and purpose:**

Temporomandibular disorders (TMD) are among the most frequent pathologies of the stomatognathic system. One problem often associated with TMD is the psychoemotional status. The aim of study was to evaluate the psychoemotional status of young adults with pain symptoms associated with TMD.

**Material and methods:**

We analyzed the data of 260 volunteers. The Research Diagnostic Criteria for Temporomandibular Disorders (RDC/TMD) form was used to diagnose TMD. The relationships between TMD/RDC clinical diagnoses and psychoemotional status, as described by the Beck's Depression Inventory (BDI) and Perceived Stress Scale (PSS‐10), were analyzed. We divide the group into four on the basis of RDC/TMD Axis I diagnosis. Group 0 included 30 students lacking TMD symptoms. Group I consisted of 30 people with myofascial pain (group IA in RDC/TMD). Group II contained 23 people with disk displacement with reduction (group IIA in RDC/TMD). Group III contained ten people (Group III diagnosis, often associated with pain).

**Results:**

We did not find statistically significant differences between the study groups. In subjects with pain (Groups I and III), we found the mean value on the BDI and PSS‐10 scales to be higher than among the pain‐free subjects (Groups 0 and II).

**Conclusion:**

In young adults with TMD accompanied by pain, psychoemotional status should also be evaluated.

## Graphical abstract

Among patients with dysfunctions of the stomatognathic system, emotional status should be considered during diagnosis. When symptoms of mental disorders occur, diagnosis and treatment should be multidisciplinary. In young adults with temporomandibular disorders accompanied by pain, psychoemotional status should be evaluated.

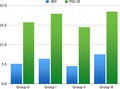

## 1 INTRODUCTION

Temporomandibular disorders (TMD) are among the most frequent pathologies of the stomatognathic system. The epidemiological studies of many researchers suggest that TMD occur in 50%–80% of the adult population, making them a disease of affluence. One concern often associated with TMD is the psychoemotional status of the patients (Manfredini, Piccotti, Ferronato, & Guarda‐Nardini, 2010; Solberg, Woo, & Houston, 1979; Vimpari, Knuuttila, Sakki, & Kivela, 1995; Yap, Tan, Chua, & Tan, 2002).

Psychoemotional state is a very frequent precipitation condition (Wieckiewicz et al., 2014; Yap, Tan, et al., 2002). The symptoms of depression include general bad mood and reduced activity, problems sleeping, and generalized pessimism. Anxiety is also an individual susceptibility and reaction to stress. It thus is responsible for introducing the organism into a state of increased arousal (Parnowski & Jernajczyk, 1977). A consequence of this is the induction of compensatory motor reactions (parafunctions). In anxiety states, the occurrence of somatization symptoms is typical—for example, a patient may complain of somatic symptoms that are not present. In terms of the oral cavity, these mainly relate to pain in the muscles of the masticatory system (Cohen, Kamarck, & Mermelstein, 1983; Korszun, Hinderstein, & Wong, 1996; Yap, Chua, & Tan, 2004).

Psychoemotional factors greatly increasing the muscular tone of the masticatory organ, including the strongest muscles of the system—the temporal and masseter muscles. Increased muscle tension becomes the cause of the pain symptoms of TMD (Stocka, Kuc, Sierpinska, Golebiewska, & Wieczorek, 2015). Other general factors, such as rheumatic diseases, endocrine brain tumors, and neoplasms in the vicinity of or within the temporomandibular joint are all very rare. TMD may also occur in the course of psoriasis, SAPHO syndrome (synovitis/arthritis, acne/acne, pustulosis/pustular psoriasis, hyperostosis/excessive bone formation, and osteitis/osteoarthritis), pain conditions of the shoulder girdle, back or head, and others (Sojka et al., 2018; Wieckiewicz et al., 2014). Current reports indicate that masticatory dysfunction injures, which significantly exceed the stomatognathic system's adaptive capacity generally arise as a result of increased tension in the masticatory muscles during parafunctions (Korszun et al., 1996; Stocka, Sierpinska, Kuc, & Golebiewska, 2018). From a clinical point of view, regardless of the severity of the disease, symptoms may occur with varying degrees of severity and frequency. TMD may manifest as a single symptom or as multiple symptoms (Yap, Chua, Dworkin, Tan, & Tan, 2002), conditioned by the individual sensitivity of the patient, which depends on individual susceptibility to stress and the threshold of pain excitability. The most frequently observed symptoms of TMD are clicking, crepitation of the temporomandibular joints, increased tension in the masseter muscles, pain in the temporomandibular joint and masticatory muscle, tension headaches, and impaired mobility of the mandible—including deviation, skipping, blocking, dislocations, and shoulder and neck pain. According to many authors (Bonjardim, Gaviao, Pereira, & Castelo, 2005; Calixtre, Gruninger, Chaves, & Oliveira, 2014; Funato, Ono, Baba, & Kudo, 2014; Gorter et al., 2008; Manfredini, Borella, Favero, Ferronato, & Guarda‐Nardini, 2010; Vimpari et al., 1995; Yap, Chua, & Hoe, 2002), parafunction symptoms may also accompany nonspecific symptoms such as a feeling of numbness in the teeth, difficulty in swallowing, burning symptoms in the throat and larynx, ear ache and auditory acoustic sensations, radiation of pain to the eye, vibrations of the lower eyelid, a feeling of great pressure in the eyeball, reduced visual acuity, ocular or orbital pain, paresthesia of the face, ears, or shoulder girdle, symptoms similar to trigeminal neuralgia, and many others. When speaking about the symptoms of functional disorders, it should be remembered that, in every advanced case of the dysfunction syndrome, increased muscle tension is observed—mainly affecting the strongest muscle.

In 1992, a group of experts in TMD treatment, created the Research Diagnostic Criteria for Temporomandibular Disorders (RDC/TMD), for the Consortium Network (Dworkin & LeResche, 1992). These criteria are based on a biased approach, taking into account, the biopsychosocial model that had previously been created. Axis I provides information leading to the assessment of TMD on the basis of a clinical trial, while Axis II consists of standard instruments based on the patient's independent opinion, which allows his or her psychoemotional state (the effect of chronic pain) to be assessed. The RDC/TMD contain a personal questionnaire consisting of 31 questions and a clinical trial form. In order to unify the test method in all centers, patient test instructions are provided. The next part contains the diagnostic algorithms for Axes I and II (Dworkin & LeResche, 1992; Osiewicz et al., 2013). The diagnostic system uses the results from the clinical trial form (Axis I) to classify patients into groups and subgroups. Group I deals with myofascial disorders and has a subgroup for myofascial pain without limited mandibular opening (group IA) and a subgroup for myofascial pain with limited mandibular opening (group IB). Group II deals with dislocation of the articular disk and has three subgroups, depending on whether the disk is unblocked (group IIA), whether there is disk lock with a limited range of mandibular opening (group IIB), or whether there is disk lock without a limited range of opening (IIC group). Group III relates to symptoms of the temporomandibular joint—specifically arthralgia (group IIIA), arthritis (group IIIB), and joint degeneration (group IIIC) (Dworkin & LeResche, 1992; Osiewicz et al., 2013).

The aim of the study was to evaluate the psychoemotional status of young adults with pain symptoms associated with temporomandibular disorders.

## 2 MATERIAL AND METHODS

The Polish version of the RDC/TMD form was used to diagnose TMD (Osiewicz, Lobbezoo, Loster, Loster, & Manfredini, 2017; Osiewicz et al., 2013). The test subjects were selected from 260 volunteers, with a mean age of 18, of both sexes, from three different high schools in Kraków. The volunteers were previously described in Loster, Osiewicz, Groch, Ryniewicz, and Wieczorek (2017). The approval of the Bioethical Committee of the Jagiellonian University No. KBET/89B/2009 to conduct the research was obtained. All students and their parents were informed of the goal of the study and signed voluntary consent for their involvement in the study. The study was conducted in accordance with the Good Clinical Practice Declaration of Helsinki.

The study's inclusion criterion was full dental arches. The exclusion criteria were malocclusions and carious destruction of dental tissues. The volunteers were divided into four groups by Axis I RDC/TMD diagnosis: There were 172 students without TMD symptoms, 52 volunteers with myofascial disorders associated with pain, 23 subjects with disk displacement, and 10 cases with arthralgia, arthritis and/or joint degeneration, associated with pain (Loster et al., 2017).

Psychosomatic state was assessed using the Beck's Depression Inventory (BDI) and the Perceived Stress Scale (PSS‐10; Beck, Ward, Mendelson, Mock, & Erbaugh, 1961; Cohen et al., 1983). The volunteers had no previous experience with the above questionnaires.

The BDI scale consists of 21 questions on the psychoemotional states experienced during the last 14 days. Subsequent response variants correspond to an increased intensity of symptoms and are scored from 0 to 3. The level of depression is calculated by summing the points. Twelve points represents a standard score (Beck et al., 1961).

The PSS‐10 scale contains 10 questions assessing the intensity of stress associated with the life situation over the last month. Before calculating the general stress intensity index, the score should be altered for four out of 10 positive questions according to the rule: 0 to 4, 1 to 3, 3 to 1, and 4 to 0. The general result of the scale is the sum of all points, yielding a theoretical distribution of 0–40. In the examined age group, the norm level was no higher than 14 points (Cohen et al., 1983). Using these tools, we examined the patients' psychoemotional status and sought a relation between pain in the stomatognathic system (a symptom of TMD) and psychoemotional status.

The examined groups were as follows: Group 0 was formed of 30 students lacking TMD symptoms (according to RDC/TMD), randomly selected from all 172 volunteers without TMD symptoms. Group I consisted of 30 people randomly selected from the 52 with myofascial disorders (RDC/TMD group IA, often associated with pain). Group II contained all 23 volunteers with disk displacement with reduction (RDC/TMD group IIA without pain), while Group III consisted of 10 people—all persons with RDC/TMD group III diagnoses (associated with pain).

We compared the relationships between TMD/RDC clinical diagnosis and the psychoemotional status, as described by the BDI and PSS‐10 scales.

### 2.1 Statistical analysis

The quantitative variables in three or more groups were compared by analysis of variance (ANOVA) when the variable had a normal distribution in the groups or by the Kruskal–Wallis test, when the normal distribution was absent. The normality of the variable distribution was examined using the Shapiro–Wilk test. A value of *p* = .05 was taken as significant. Analysis was carried out in the R program (version 3.3.3).

## 3 RESULTS

Using simple individual sampling for the analysis, a group of 93 participants was selected. Group 0 was formed of 30 students lacking TMD symptoms (according to RDC/TMD), randomly selected from all 172 volunteers without TMD symptoms. Group I consisted of 30 people randomly selected from the 52 with myofascial disorders (RDC/TMD group IA, often associated with pain). Group II contained all 23 volunteers with disk displacement with reduction (RDC/TMD group IIA, without pain), while Group III consisted of all 10 volunteers with arthralgia, arthritis, and/or joint degeneration (RDC/TMD group III, associated with pain).

**Figure 1:**
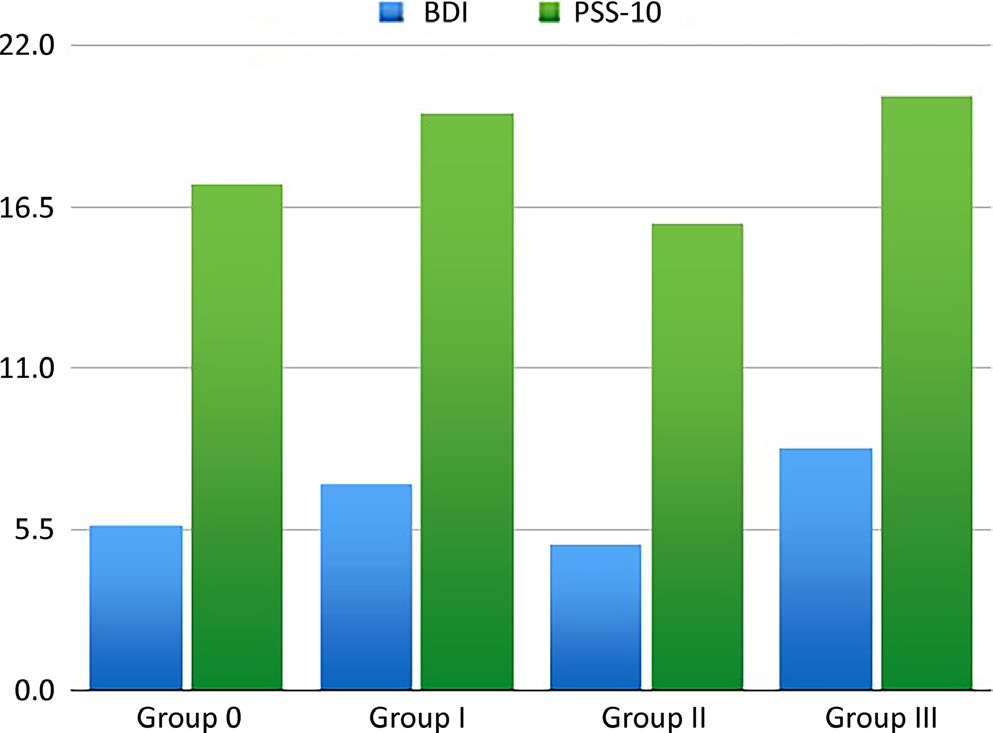
Graph of the mean Beck's Depression Inventory (BDI) and PSS‐10 scores among subjects without TMD (Group 0) and with various forms of TMD (Groups I, II, and III). Axis I: Group 0: control group; Group I: those with myofascial disorders with pain; Group II: those with dislocation of the articular disk without pain; and Group III: other joint conditions with pain

Among the controls (Group 0), the average score on the BDI was 5.63 (ranging from 0 to 19); the range on PSS‐10 was 10–17.17 (with a minimum of 8 and a maximum of 32). The average BDI and PSS‐10 scores were respectively 7.07 (0–19) and 19.7 (9–30) in Group I, 4.96 (0–13) and 15.96 (5–24) in Group II, and 8.3 (0–19) and 20.3 (5–30) in Group III. The average BDI did not exceed the accepted standard, while the PSS‐10 scale was exceeded in all groups. There were no statistically significant differences between the study groups. In those subjects with facial pain (Groups I and III), we found the mean value on the BDI and PSS‐10 scales to be higher than among the pain‐free subjects (Groups 0 and II). The BDI and PSS‐10 results for all the groups are shown in Figure 1.

## 4 DISCUSSION

In a sample of young adult volunteers, our research compared results from Axis I RDC/TMD with those of two surveys of psychological status (the BDI and PSS‐10 scales). The results of these two tools gave similar results regarding the psychological status of the subjects. We then divided the subjects into four groups on the basis of their RDC/TMD Axis I diagnoses. Group 0 represented those free of TMD symptoms and as Group II without pain, and Group I and III those respectively associated with pain. To obtain similarly sized groups, we randomly selected 30 subjects from the 172 healthy nonpatient cases to form Group 0, 30 from 52 subjects to form Group I, and all volunteers who matched the criteria for Groups II and III. We took into account the presence of pain, in Groups I and III, and it was reflected in the psychological status.

Data from the literature show that depression is common among patients with complaints of masticatory muscle pain; these often coexist with general health problems (Bonjardim et al., 2005; Dabrowski et al., 2010; Garimella, Sears, & Gehi, 2016; Gomaa, Elmagd, Elbadry, & Kader, 2014; Korszun et al., 1996; Manfredini, Borella, et al., 2010; Minghelli, Morgado, & Caro, 2014; Sojka et al., 2018; Stocka et al., 2018; Zielinska‐Blizniewska & Olszewski, 2009). In our examined groups with facial pain (Groups I and III), we found the mean value on the BDI and PSS‐10 scales to be higher than among the pain‐free subjects (Groups 0 and II). This confirms that physical pain plays an important role in psychoemotional conditions. Several articles have already attempted to explain the correlation between TMD and psychological distress (Stocka et al., 2015, 2018).

In a study from the Department of Prosthodontics, University of Damascus, which aimed to find a correlation between stress and TMD in a Syrian population, statistically significant differences were found between patients and a control group in three psychosocial stress surveys (Salameh, Alshaarani, Hamed, & Nassar, 2015). That study involved supplementary analysis of the level of cortisol in the saliva, to answer the question of whether the level of cortisol was correlated with the results of the PSS‐10, contrasting patients with TMD and with those in the control group; RDC/TMD axis II was used. The differences in level of cortisol in the saliva between the TMD cases and the control group proved to be statistically significant. No statistically significant differences were found in the level of cortisol between the TMD groups. This means that subtypes of patients with TMD have similar levels of stress. Those results are similar to our observations, with no statistical differences being found between the stress levels of the TMD patients.

A study from the University of Sarajevo described the level of salivary cortisol in students with chronic myofascial pain (MFP) and investigated its relationship with TMD during oral examinations. The correlation between salivary cortisol concentration, TMD‐associated MFP, anxiety, symptoms of depression, somatization, and stress was also analyzed (Božović, Ivkovic, Račić, & Ristić, 2018). The study was included 60 students divided into two groups: The first contained those diagnosed with MFP (*n* = 30) and second group contained healthy students (*n* = 30). The level of cortisol in the saliva was measured on the day of an oral examination and during a control day, when students were not taking examinations. Symptoms of depression, somatization, perceived stress, and anxiety were diagnosed in accordance with RDC/TMD Axis II, the Perceived Stress Scale. It was shown that levels of salivary cortisol were significantly higher in the group of MFP students in all phases of the measurements (on the day of the examination and the control day) compared with the control group (*p* < .01). Students with MFP also showed significantly higher values for symptoms of depression, somatization, and anxiety than did the control group. There were no significant differences between the groups in terms of their scores for state of anxiety and stress experienced. It was found that the level of cortisol in saliva correlated with symptoms of depression, anxiety, and stress, but not with chronic pain, somatization, or anxiety traits in students with TMD. Thus, the concentration of cortisol in saliva may be an important indicator of mental disorders in TMD.

A cross‐sectional observational study conducted at the University of Brazil involved a group of volunteer students with an average age of 24 ± 7 years. Its aim was to evaluate the prevalence of TMD and its association with perceived stress and common mental disorders (Augusto, Perina, Penha, Dos Santos, & Oliveira, 2016). To evaluate TMD, they used Fonesca's Anamnestic Index. Stress was measured with the Self Reporting Questionnaire (SRQ‐20) and PSS‐14. The study group consisted of 586 students. TMD signs and symptoms were found in 71.9% and significant correlations were seen between TMD and perceived stress (PSS); there were moderate correlations between TMD and common mental disorder, and also between TMD and parafunctions habits. These results thus clearly show an association between TMD and stress. The association is clearer than in our study as we used volunteers with an average age of 18, while the Brazilian study group was on average 24 ± 7 years old; stress had thus affected the stomatognathic system for longer.

The aim of systematic review by Wieckiewicz, Zietek, Smardz, Zenczak‐Wieckiewicz, and Grychowska (2017) was to examine the relationship between masticatory muscle pain (MMP) and mental status. These authors indicated that, in the light of the literature, the relationship between mental status and masticatory pain (a symptom of TMD) has still not been clearly established. This does not mean that the relationship will be not found when other signs and symptoms of TMD are taken into consideration.

According to the WHO definition, health is “a state of full physical, mental and social well‐being, not just a complete absence of disease or disability.” Functional disorders of the masticatory muscle system, on account of their prevalence, can be counted as diseases of society and of civilization (Loster et al., 2017; Sojka et al., 2018; Vimpari et al., 1995; Yap, Tan, et al., 2002). Temporomandibular dysfunction affects very many people, presenting as headache, temporomandibular joint pain, and clicking, and other symptoms. These can significantly affect the well‐being and quality of life of people suffering from them. Differences in results between surveys may result from study limitations, such as differences in research procedure, the number of respondents, and socio‐economic factors.

In the available publications, a number of authors (Augusto et al., 2016; Božović et al., 2018; Gorter et al., 2008; Salameh et al., 2015; Wieckiewicz et al., 2017) have shown that young adults are at a high risk of exposure to stress, which can affect the status of the stomatognathic system. Young adults live under constant daily stress, such as that resulting from examinations, own expectations, family expectations, lack of sleep, and limited free time. Another important aspect is that during puberty many changes occur in the body, including hormonal changes and mental maturation. These can have a significant impact on reducing overall well‐being, including health and thus psychoemotional status. Among patients with dysfunctions of the stomatognathic system, emotional status should thus be considered during diagnosis. When symptoms of mental disorders occur, diagnosis and treatment should be multidisciplinary. We still need further studies on a larger group if we are to better understand the connection between psychoemotional status and TMD.

## 5 CONCLUSIONS

In young adults with TMD disorders accompanied by pain, psychoemotional status should be evaluated.

## CONFLICT OF INTEREST

All the authors stated no conflict of interest.

## Data Availability

Data available on request from the authors.
